# Respite: carers’ experiences and perceptions of respite at home

**DOI:** 10.1186/1471-2318-12-42

**Published:** 2012-08-03

**Authors:** Nan Greenwood, Ruth Habibi, Ann Mackenzie

**Affiliations:** 1 Faculty of Health and Social Care Sciences, St George’s University of London and Kingston University, 2nd Floor Grosvenor Wing, London SW17 0RE, UK

## Abstract

**Background:**

Informal carers play an important role in supporting people with long-term conditions living at home. However, the caring role is known to have adverse effects on carers such as poorer emotional health and social isolation. A variety of types of respite may be offered to carers but little is known about the benefits of respite, carers’ experiences with it, or their perceptions of care workers. This study therefore investigated these experiences and perceptions.

**Method:**

Recorded, semi-structured interviews were undertaken with twelve carers receiving weekly four-hourly respite. Carers were either caring for a person over sixty or were over sixty themselves. Interviews were analysed thematically.

**Results and Discussion:**

Respite sometimes alleviated carers’ constant sense of responsibility for their cared for. Trust, whether in the service provider or individual care workers, was essential. Carers lacking this trust tended to perceive respite as less beneficial. Low expectations were common with carers often unwilling to find fault. Care workers were frequently seen as very kind with some carers valuing their company. Care workers who were flexible, communicated well and responded to the cared for’s needs were valued. Stimulation of the cared for during respite was very important to most carers but the perceived benefits for carers were often very individual. Many carers used respite to catch up with routine, domestic tasks, rarely using it to socialise.

**Conclusions:**

For many carers, respite was a way of maintaining normality in often difficult, restricted lives. Respite allowed continuation of what most people take for granted. Carers frequently viewed respite as intended to improve their cared for’s quality of life, rather than their own. This centrality of the cared for means that carers can only really benefit from respite if the cared for is happy and also seen to benefit. Future research should investigate the perspectives of carers and their cared for, focussing on different demographic groups by features such as age, gender, ethnicity and diagnostic groups. However, without greater clarity about what respite is intended to achieve, clear evidence of a positive impact of this intervention may remain difficult to identify.

## Background

### Carers

Awareness of the numbers of carers (also known as informal carers or caregivers) and the vital role they play in supporting people with long-term conditions is growing.

In the UK over half of carers are women (58%) and approximately 1.2 million carers care for over 50 hours a week [1].

Carers can be defined as someone who provides:

‘… unpaid care by looking after an ill, frail or disabled family member, friend or partner.’ [1: p1]

The number of carers and the roles they play in supporting people with long-term conditions is likely to increase as the population ages and more people survive longer with disabling conditions. According to the Office of National Statistics (ONS), by 2033, there are expected to be over three million people aged over 85 years making up five percent of the UK population and nearly a quarter of the total population will be 65 or older [2]. Many of these people will be supported by a spouse, adult child or both.

While it is now acknowledged that carers often gain satisfaction from their caring role, for example [3-7], it is also well recognised that being a carer can be stressful and may adversely affect carers’ social networks and physical and emotional health [8]. Especially in the long-term, social isolation amongst carers is frequently highlighted, for example [9,10]. Carers often report high levels of stress, depression and anxiety as well as physical health problems [11]. They also sometimes describe family conflict and negative feelings such as anger and frustration [12].

### Respite

Given the sometimes difficult nature of their role, it is very important that carers are offered support to help them maintain it and reduce any adverse effects of caring. Respite is one such form of support. It refers to a range of services including day-care, institutional respite and respite at home (in-home respite) and is often offered as part of a package of care but may also be provided on an informal basis [13]. It can be defined as:

‘… an arrangement to allow caregivers relief or ‘time-out’ from their care commitments, which may be provided on a regular basis or in emergencies.’ [12: p298].

Providing carers with respite recognises that they may need time to rest and be away from their caring responsibilities [14,15]. It has also been argued that respite helps the carer continue caring and may delay nursing home placement [16]. Initially respite was regarded solely as a break for carers but it is now recognised that respite should at least be framed around the needs and wishes of the cared for [17] and to be successful respite should be a positive experience for the cared for as well as the carer [14]. Indeed the cared for may benefit from time apart from their carer [12] and respite can give the cared for increased opportunities for a greater range of activities, greater independence and improved quality of life [18].

Arksey et al. [19] reviewed eight studies investigating respite for carers of people with dementia. They concluded that carers frequently reported high levels of satisfaction with in-home respite. In the studies investigating reasons why carers were satisfied, it was often linked to their perceptions of the quality of the care provided and benefits for their cared for.

Research has sometimes used quantitative methods to investigate the impact of respite on carers, for example in terms of health and well-being, but there is little evidence that respite in general has either a consistent or enduring beneficial effect on carers’ well-being [19,20]. Both the effectiveness and cost-effectiveness of respite remain poorly understood [21]. However, the diversity in the precise nature of respite and of the client groups makes judgements about the impact of specific forms of respite difficult.

In their review of research investigating respite for the frail elderly, Mason et al. [21] concluded that overall for all types of respite, carer satisfaction was generally high but the impact was small with modest benefits for some sub-groups. According to these authors the evidence suggests that respite for carers of frail elderly people generally has a small effect on carer burden and physical and mental health. However, due to the nature of the research, it was not possible to draw firm conclusions on respite’s effectiveness. The authors suggested that respite needed to be flexible and responsive to the needs of carers and their cared for and to any changes in needs over time.

As part of a systematic review of a variety of types of interventions for carers, Victor [22] summarised studies investigating a range of forms of respite and reached more positive conclusions than Mason et al. [21]. Victor identified four studies evaluating respite services that she described as a ‘sitting service’. Respite here involved a care worker providing care within the home and effectively replacing the carer for a period of a few hours [23-26] cited in [22]. All four identified studies included carers of elderly people, with two specifically for people with dementia [25,26]. Using quantitative methods, Harper et al. [23] identified improvements in carers’ emotional well-being after three months of receiving the service. However, there was huge variability with well-being declining in some carers. These authors concluded this may have been related to deterioration in the cared for’s condition. Also using quantitative methods, Milne et al. [24] reported reduced carer strain over time for those receiving the sitting service compared with increased strain amongst carers who had chosen not to receive the service. However, the difference was not statistically significant. O’Donovan [25] used a structured evaluation and found that carers said that respite had given them ‘peace of mind’. The only qualitative evaluation reviewed here [26] also reported that participants said respite brought them peace of mind, allowing them to relax and worry less.

Overall looking at all types of carer breaks, Victor [22] concluded that they give carers a chance to rest both emotionally and physically and to catch up with everyday tasks, social activities and sometimes employment. In some cases breaks can be critical in allowing carers to continue caring. However, the impact can be complex. On the one hand, breaks may give carers a sense of normality, freedom and relief but may also lead to feelings of guilt and anxiety. In addition, similar to Mason et al. [21], this review stressed that respite must be acceptable to the cared for, tailored to the situation and flexible.

There is little published qualitative research investigating carers’ perceptions of respite. In one study carers caring for people with a range of disabilities were asked for their views on a variety of types of respite [27]. These carers saw respite as a service that provided a sense of ‘freedom’ and ‘normality’. They valued home-sitting services particularly highly. A qualitative evaluation of a domiciliary respite service for carers of younger people with dementia [28] reported that carers were very satisfied with the respite service and rather than using the time for recreational or social activities, more frequently caught up with household chores and shopping. Based on individual need, care workers undertook three main activities: practical (such as help with dressing), therapeutic (for example, stimulating and entertaining) and educational activities where the carers learnt coping skills. In particular, carers valued the personality, experience and flexibility of care workers. In terms of the effects on the person with dementia, they talked about the care worker helping to keep things going as usual. Overall respite ‘made a difference to carers’ lives mainly because they could leave their relative for short periods knowing that he or she was safe and cared for.’ [29: p382].

The respite under investigation here is in-home respite where a care worker comes to the family home and either stays with the cared for or takes them out. This form of respite has several possible advantages in that the cared for person can remain in familiar surroundings and does not necessarily have to be made ready to take out. It has been argued that in-home respite can facilitate ‘the rhythms of family life’ [29] cited in [19]. However, there can be initial reservations about letting a ‘stranger’ into their homes [28].

Throughout this article we refer to the informal, unpaid carer as the ‘carer’; the person being looked after is referred to as the ‘cared for’ and the paid individual coming into the home to look after the cared for is referred to as the ‘care worker’. These terms are used for consistency but it is recognised that these terms have their limitations and may not always be the same as those used in other literature.

### Aims

In response to the paucity of qualitative literature investigating home based respite, the aims of the study were to investigate carers’ experiences of in-home respite, their perceptions of care workers and their perceptions of the impact of respite on themselves and their cared for.

### Study approvals

Both the National Research Ethics Committee and the South West London Research Ethics Committee were provided with details of the study and both said that research ethics approval was not required as the study was an evaluation as opposed to research. However, ethical principles were followed, confidentiality was assured and informed consent was always obtained.

### The respite service

The respite service was offered to carers in a South West London borough who were caring for someone at home with an identified health need. Respite was provided for a maximum of four hours per week by a third sector organisation who recruit, train and pay care workers to stay with the cared for.

## Method

### Recruitment

Potential carer participants were contacted by the organisation providing respite and asked if they would be willing to be interviewed. It was stressed that there was no obligation to participate and that confidentiality would be maintained. If carers agreed, the research team was provided with their telephone numbers. A researcher (RH) then approached carers and confirmed whether they were still happy to participate in the study. Interviews were arranged at a time and place convenient for carers.

To be included, carers had to be either currently receiving respite or to have been receiving it until very recently; to have had the service for a minimum of three months and either to be aged over 60 years themselves or to be caring for someone aged over 60 years. One carer who was receiving respite was not invited to participate in the study as the service provider felt that she should not be contacted because of particularly difficult circumstances.

### Interviews

With the carers’ permission interviews were audio recorded. The interviewers (RH and NG) both have considerable experience in recruiting and interviewing carers and older people. A semi-structured approach with a topic guide containing open ended questions was used. This method defines areas to be explored but also allows both the interviewer and interviewee to diverge from the main topics in order to pursue an idea in more detail [30]. Areas covered included carers’ experiences with the respite service and their perceptions of the impact of respite on themselves and their cared for. Carers were also asked to provide the following information: carer and cared for gender, age, ethnicity, relationship, length of caring and the cared for’s diagnosis.

### Analysis

Interviews were transcribed verbatim and then analysed by hand to identify themes in the interviews. Thematic analysis is ‘a method for identifying, analysing, and reporting patterns (themes) within data’ [31]: p6]. Once the interviews were transcribed, two researchers independently immersed and familiarised themselves with the data, reading and re-reading a selection of transcripts. They then began to generate initial codes from the data. This approach reduces the data. Codes are then grouped into broader themes allowing the interviews to be described and summarised. The researchers then came together and discussed the themes until consensus was reached between the researchers and the data could be reduced no further [32]. The remaining transcripts were then analysed using the agreed themes. During the analysis, attention was paid to the characteristics of carers (for example gender) and the carers’ situations (for example the diagnoses of the person they care for) in order to determine whether these had detectable relationships with the themes identified.

### Findings

Carer participants in this study were all regularly providing care for a relative who had identified health needs. All were receiving the maximum of four hours respite per week.

Thirteen eligible carers were identified by the service provider but the researcher was unable to contact one carer. Twelve carers were interviewed. All interviews took place in carers’ homes. In seven interviews the cared for were also present but in most cases they did not participate in the interview.

On average interviews lasted approximately half an hour with the longest lasting one hour.

### Participants

Carer background details can be found in Table1. All the carers were either themselves aged over 60 years or were caring for an older person. Three quarters were female (76.9%) and most were White British (69.2%) and over 60 years old (69.2%). The vast majority were family members, usually spouses or adult children caring for a parent. One carer described herself as a friend. All carers said they had been carers for more than a year and nearly half had been in the role for more than five years (46.2%).

**Table 1:** Carer demographics

**n = 12**	**n (%)**
**Gender**	
Female	9(75.0%)
Male	3(25%)
**Relationship to cared for**	
Spouse	5(41.7%)
Adult child	3(25.0%)
Parent	1(8.3%)
Other (grandchild, sibling and friend)	3(25.0%)
**Age in categories (years)**	
41-50	3(25%)
61-70	2(16.7%)
71-80	6(50.0%)
81-90	1(8.3%)
**Ethnic group**	
White British	9(75.0%)
Black British	2(16.7%)
Other	1(8.3%)
**Length caring (years)**	
1-2	3(25.0%)
2-3	1(8.3%)
3-4	2(16.7%)
4-5	1(8.3%)
5-6	2(16.7%)
> 6	3(25.0%)

The cared for were slightly older than their carers and were mostly diagnosed with age-related conditions such as dementia, stroke and Parkinson’s disease (Table2).

**Table 2:** Cared for demographics

**n = 12**	**n (%)**
**Gender**	
Female	7(58.3%)
Male	5(41.7%)
**Diagnosis**	
Dementia	8(66.7%)
Other (including stroke, Parkinson’s disease, physical illness, and depression)	4(33.3%)
**Age in categories (years)**	
41-50	1(8.3%)
61-70	1(8.3%)
71-80	5(41.7%)
81-90	4(33.3%)
90+	1(8.3%)
**Ethnic group**	
White British	6(50.0%)
Black British	3(25.0%)
White European	2(16.7%)
Other	1(8.3%)

The length of time receiving respite varied from three to 13 months. The mean was 7.6 months and the median 6.5 months. Most carers were still in receipt of respite but one had stopped because their cared for had moved into an institution.

Transcribed interviews were analysed and the following themes were identified. Quotes are included to demonstrate how the themes were derived. Where necessary the care worker and the cared for are assigned initials instead of names. To ensure anonymity, only the relationship of the carer to the cared for is provided. Place names have also been removed.

### Themes

A number of themes were identified and these have been grouped according to the main aims of the study and the context of being a carer. Overall there was remarkable consistency in the themes despite differences in individual carer characteristics and circumstances such as ethnicity and diagnosis of the cared for.

### Context of being a carer

#### Constant responsibility

Carers described a constant, unrelenting and, at times, overwhelming sense of responsibility for their cared for, often making it difficult to leave them. A few vividly described their guilt if they left the cared for alone for even a few minutes. Sometimes this feeling of total responsibility was because the cared for needed continuous attention for safety reasons but for others it was because the cared for wanted to know exactly what the carer was doing.

*‘… I cannot leave him for a minute and I actually can’t, you know, if I go upstairs … I can hear him in a minute. You know he starts calling – even if I say I’m just going up to get something he starts worrying… it’s pretty full on you know.’* Wife

For some carers, respite was the only time when they felt that they did not have to hurry back. It offered the opportunity for carers to relax as someone else had taken on this responsibility

*‘.....That’s why it make me tired sometimes. I can’t even relax for five minutes. Every five minutes to look at the clock and then I’m rushing to give him his dinner…* [With respite] *I feel relaxed. And just sometimes sit, or do shopping.’* Wife

The participants also sometimes described an overarching sense of tension which only dissipated when they had full confidence in the care worker. This confidence was not always there when they first met the care worker but usually grew with time. Even then, some carers felt pressure to return very promptly, or even early as the carer worried about making the care worker late.

*‘As soon as the care worker comes I’m ready. I have a friend who used to live a couple of doors away, moved away. And she comes over and we get in the car and go … And we stay out, you know … but I’m never late, because I’m thinking of the care worker - she’s got to go.’* Wife"

One carer summed up the feelings of many carers – the sense that caring would last a very long time but also had a downward trajectory.

*‘When you’re a carer you know that someone is going to become worse, or remain the same for a long time. And the knowledge of it never changing, or becoming worse is a massive matter, because it’s about loss. You’ve lost the person you had, they’ve lost their independence, you know, and they can see you distressed as well, which you’re always conscious of as a carer, so um, it’s a bit tricky.’* Daughter"

#### Trust

Trust played a critical role in accepting respite. Trust related both to trust in the respite service as a whole and in individual care workers. Carers had to have overall confidence in the service provider in order to even consider accepting respite but they also had to trust the individual care worker to look after their cared for and be unsupervised in their homes. Trust in the individual care workers is essential. Without it, carers feel unable to leave their cared for over any length of time and unable to enjoy and benefit from their time away. Those carers who lacked confidence in the care worker tended to either stay at home, perhaps in a different room, or to stay out for only short periods and to worry constantly while they were out. Trust in the care worker included characteristics such as their sensitivity to the cared for’s needs and reliability. Unreliable care workers meant carers were unable to be sure the care worker would arrive when expected making them unable to plan ahead. As a result they were unable to take full advantage of respite.

Participants often assumed that the respite provider would provide a good service because it had been recommended by a trustworthy source such as Age UK. However, sometimes because of poor past experiences with paid carers, participants initially had low expectations and uncertainty about the care worker.

*‘This is regular whereas … whenever I rang them* [previous care agency] *they weren’t available. And when they did come, the person they sent was very um, er, I suppose forthright, but in a kind of an inappropriate way, and I didn’t really like to leave her with my Mum. Whereas your, the person that we get regularly now is great.’* Daughter

#### Carers’ low expectations and powerlessness

Another theme in the interviews related to carers’ apparent feelings of powerlessness and low expectations of support. Not only were carers often unwilling to ask for help from family and friends but they also were generally unwilling to complain about the respite. Most carers expressed satisfaction with the service but some were not totally happy, for example, with the care worker. These carers often said they would not want to complain.

*‘… the only thing I think sometimes is maybe she could have helped me press my girl’s clothes you know. I never asked her but apparently she will do it if I ask her. Yes but it’s alright, I can’t complain.’* Husband"

*‘I mean I don’t know if there would be anybody better, you know, who would communicate.’* Wife

Many appeared to believe that they were lucky to receive any help at all and therefore should neither criticise the care worker nor ask them to do additional tasks.

*‘If somebody’s been kind enough to offer you help, you don’t want to throw it back at them.’* Friend

*‘Yeah. I’m sure she would be quite willing, and they’re well trained so I’m sure she could do it, but I just think it’s a little bit much to ask.’ S*on

Complaining may have been harder for carers because they were unwilling to upset the care workers since they regarded them as very kind, pleasant people.

*‘And then they put me on to one girl who was not suitable. She was very nice, there was nothing wrong with her at all, but she did not connect with her* [cared for] *in any way.’* Friend

This tendency to accept the situation, even if carers were unhappy with it, means that the potential positive impact of respite is not being maximised.

### Experience of respite

#### What carers do during respite

Carers described a range of activities in their four hours of respite but socialising or going out purely for recreational purposes were rare. It was perhaps surprising how frequently respite was used to do mundane, everyday tasks. Sometimes they remained at home and caught up on chores such as cooking, particularly if they were unable to do this when with the cared for. Many carers also used respite as an opportunity to go shopping or have medical appointments.

*‘Well shopping, or whatever we have to… my husband has these, has to go to the doctor quite often and things like that, or whatever. You know. We carry on. I might be doing a whole load of washing or something… Yeah… Daily things, yes… it really is a tremendous help for us.’* Friend

Some carers did not stay out for the full four hours of respite and some preferred, at least sometimes, to stay at home.

*‘I’m so exhausted sometimes that I don’t want to go out.’* Wife

A few did use the time for recreational activities.

*‘… it’s good to be able to go to a movie or something… I did go and see a film – it just happened to work. It started at 8.15 and was finished by 10.15. So fantastic.’* Wife

#### Benefits for the carer

Carers often spontaneously mentioned respite’s positive effects but they were also directly asked if they could think of any ways they might have benefitted from the service. Carers used words like ‘vital’ and ‘God send’. One daughter expressed this very strongly:

*‘I would just say – given me my life back and maintained my sanity. Because you need that out time, you really do. Yeah otherwise you’ll just go crazy*… *otherwise I would explode.’* Daughter

*‘I don’t know what I’d have done without them. The few weeks that I’ve had them, because it’s given me a break.’* Wife

*‘It’s four hours where I can go and enjoy myself.’* Wife

Some carers described respite not only as a break from caring but also as a means of literally ‘getting out’ of the house.

*‘Um, a bit of relief I think. You know, sort of, to get out of these four walls and just to get away for a couple of hours.’* Wife

Just by leaving the house, respite could therefore be enjoyable and relaxing and could reduce the stress and isolation of caring.

*‘Yes you need to – it’s terribly easy to become isolated – not, you know emotionally isolated – you know, you think I don’t want to go out because you’re so used to not going out - it’s very easy to become a sort of stick in the mud and not get stimulated…’* Wife

For some carers respite was valued because it meant that they could go out without having to take their cared for with them. This not only made life easier for carers but was also better for the cared for. It was striking how often carers used the time to do everyday chores but it is important to note that carers often took pleasure in the fact that they could do these chores without the cared for, whilst also leaving their caring responsibilities behind. One wife said:

*‘… you know even if I went down to the supermarket, you know, and tootled around – you know I can go and do some shopping and I don’t know what time the shops in the mall shut but you can go and just do bits and bobs and it’s just nice not to feel always rushing.’* Wife

*‘You feel, it just puts me at ease that someone, someone’s sitting with her.’* Grandchild

However, there were other less obvious benefits for some carers. Several enjoyed the opportunity to chat to the care worker and appreciated someone simply listening to them. One carer even suggested that it was a pity that she felt she should leave the house.

*‘Yes, but as I say it was a shame really because it was a nice break for me to talk to her to be honest because, you know, when they’ve got dementia you don’t get proper conversation – it’s a mixed up, wandering conversation, you know, but no… no she was very good*… *Yes it was a kind of – how can I put it? You could chat about all sorts and I would. I didn’t go out straightaway unless I had to, or if I was messing about upstairs doing something and I’d come down to make a cup of tea or something then… you enjoyed the chat – she was so understanding and it was great, that was nice, you know.’* Sibling

*‘I’ll have a cup of tea before I go and I’ll talk about the kids and this and that.’* Wife

The fact that the respite was there and at a regular time allowed carers to plan and spurred them into taking advantage of the break.

*‘No it does and it sort of galvanises you into, you know, maybe climbing out of your jeans. And thinking can I talk about anything, except what I was eating. Or ‘Has he fallen over recently?’ No, no it does make you switch on to what is going on in the world a bit.’* Wife

Respite was sometimes seen as benefitting the cared for which could indirectly also have a positive impact on the carer. One carer commented that the care worker gave the cared for something else to think and talk about which had benefitted their relationship.

Ironically respite, although offering relief, also gives carers time to think about their situation which could be difficult but had helped one carer with her overall adjustment.

*‘… because when you’ve got time to wind down, you’ve also got time to get upset, but it gives you time to adjust to what’s happening to you. I can’t explain, but it’s just. ‘Cause you’re so busy, when you’re busy, busy, busy, busy, concentrating on doing practical things, the good thing is you don’t think, the bad thing is you’re not relaxing. So when you get a chance to relax, you then get a chance to reflect, and that can make you very sad. But it’s also necessary, for you to mentally adjust to what’s going on… But I, I go through it all the time. It’s constant… I think it’s just very important to have that time away.’* Daughter

### The cared for

#### The centrality of the cared for

Although respite is ostensibly offered to benefit the carer, the cared for must also be happy with it. Respite must suit the cared for and fit in with their needs. If it does not, some carers then stop the service.

A minority of carers were unhappy with the service usually because the care worker did not suit the person they cared for. The cared for needs to feel comfortable with individual care workers. Where the cared for and care worker did not get on well or did not communicate well, carers were unhappy about leaving them. This means that respite is likely to be of little benefit for the carer.

One wife said that her ideal care worker would *‘do what I do’.* She was very unhappy with the care worker.

*‘… she has nothing else to do and then all she does is sit with him. You know what I mean she … doesn’t really touch him or communicate in any way whatsoever. She doesn’t seem to have that sort of kindness to touch him and all that sort of thing, so that’s the sadness of it.’* Wife

She felt that during respite her husband was *‘left isolated in his own little world’.*

This centrality of the cared for was also clear when carers talked about the sorts of activities undertaken by care workers during respite.

#### Benefits for the cared for

For many carers the positive impact of respite on their cared for appeared more important than any benefits for themselves. Stimulation was the most commonly mentioned benefit for the cared for. This included taking them out on trips or simply giving them someone different to talk to. As a result of their conditions, many of the cared for seldom left the house and were socially isolated. Here the care worker was seen as valuable in providing a ‘new face’ for them.

*‘It’s having other stuff, even if they don’t do very much, at least it’s, you know, a change.’* Son

*‘And I mean I’ve heard H’s stories so many times - I was there anyway – the evacuees and – I wasn’t in the er – I was three years younger than her, but the stories, you know, it’s repetitive – ‘Oh not again’- you know – so it was good for H because she could talk to people about it and they didn’t say 'Oh I’ve heard that before’.’* Sister

*‘And playing cards with her and stuff like that. It makes her mind work more. Instead of just sitting there doing nothing.’* Grandchild

Someone who could focus their attention solely on the cared for was also valued.

*‘I don’t have time, we have lots of grandchildren and I have my husband to look after, and you know, ourselves. And I don’t have time to entertain her or take her out shopping. If we go shopping… it takes absolutely forever. And the care worker comes and goes shopping with her and takes her out and does things…Well, it means a lot to us, because it gets her out and about and doing things that she perhaps might not do otherwise… think it’s a wonderful service.’* Friend

In two situations, the care worker was perceived as the main catalyst to improving the cared for’s behaviour and mood. In one case, the cared for had now started going out alone, something he had not done for a long time.

*‘… and what I’ve noticed since the care worker started coming is that he actually gets up and the first couple of months he wasn’t too good – I had to say, J is coming, you need to be up, you have to be ready … Yes* [now] *he’s up and ready.’* Mother

One daughter put her perception of respite being primarily to benefit her mother very clearly:

*‘And I, I don’t only see it as respite. … I see it as befriending of my Mum.’* Daughter

### Care workers

#### What care workers do during respite

Generally carers felt that care workers fitted in with what both carers and the cared for wanted. Often all that was required was for the care worker to sit and keep the cared for company by chatting, reading to them and listening to them. Although not expecting it, one wife was very appreciative of the support she had received from the care worker who had done some housework and had also called the council to get her regular help with housework.

However, carers emphasised that whatever the care worker usually did, had to reflect and be responsive to the cared for’s needs and wants.

*‘She brings her slippers and makes herself at home, and sits and chats with my husband and if he wants to have a sleep, she sits and watches him.’* Wife

*‘Yeah, yeah, that’s right. And using magazines and images. You know she has even brought her own magazines in sometimes … To chat to Mum, um, it’s really, really nice.’* Daughter

A husband really appreciated the fact that the care worker appeared willing to do anything around the house as well as care for his wife allowing him to relax and to reduce the strain he felt.

Other care workers took the cared for out. This was particularly valued by some carers because it stimulated the cared for. Two carers thought this had been a vital element in the improvement in the cared for’s condition.

*‘… and takes her on visits, yes. Which is excellent… we had the mental health nurse here yesterday, who asked her lots of questions, which she answered much better this time than she has done normally.’* Friend

#### Characteristics of good care workers

A clear picture emerged of the qualities of a good respite care worker. The frequent references to them as ‘*lovely, kind’* people emphasised the centrality of this trait.

The following quotes cover most of the characteristics carers highlighted. Some of these relate to personal characteristics, some to training and some to the past experiences of care workers. There were frequent references to the necessity of communicating well, being on the ‘*same wavelength’,* being adaptable and stimulating the cared for.

*‘Someone who can listen to her, and give her the time. That’s the main two things. And communication.’* Grandchild

*‘Um…no. I mean, I think she is quite… she’s taken phone calls for me from the hospital, or messages. No she seems quite confident, doesn’t she? Very confident person.’* Wife

According to one wife, care workers should *‘bring the sunshine and chat into the house.’*

Other important features included fitting in with and being sensitive to the cared for’s needs and being responsive to any changes.

*‘I think they’ve got to be quite, quite adaptable. ‘Cause, um, on the whole, my mother is in quite good spirits, but sometimes she, you know, and they’ve got to be able to key in to that, to be quite, aware whether to talk a lot, or to not.’* Son

Some carers highlighted the importance of relevant experience or intuitively responding appropriately.

*‘She’s very chatty… she can almost have a conversation with herself, and actually that’s what you need with someone with my Mum’s communication difficulties. And it’s, a lot of other people don’t have that, you know, they feel uncomfortable not having a response, not having a two way conversation… although there may be people who haven’t had that experience, they just instinctively… they’re OK about that.’* Daughter

## Discussion

Respite at home was perceived by these carers as providing a short time away from their caring role and was often regarded as a way of maintaining normality, allowing them to perform every day, routine tasks. Rather than being seen as focussed around the carers’ needs, it was often seen as a service for the cared for and a means of increasing the cared for’s quality of life. Respite was seldom used by carers to engage in social activities but more often it was a way of accomplishing domestic chores without the presence of the cared for. Thus respite served as a brief escape from the unremitting nature of the carers’ role. Guilt at leaving the cared for was seldom described but some carers clearly did not want to go out for long periods. Indeed some preferred to remain and take advantage of the company of the care worker.

Carers often saw respite as a way of providing stimulation for the cared for and introducing someone else into the cared for’s restricted lives. In this sense care workers provided something that the carers themselves could not offer. Lively care workers who communicated well with the cared for were particularly valued. The importance of this fit between the cared for and the care worker cannot be underestimated. Without this, the carer is likely to be both less comfortable with leaving the cared for and less likely to benefit from respite. Indeed this focus on the cared for was emphasised by the fact that carers stressed that respite had to be flexible and framed around the cared for’s possibly changing needs. Our findings here echo research from elsewhere [17,18,21,22].

As long as carers trusted the care worker, respite gave them peace of mind and the chance to relax [22] with an opportunity to relinquish some of their responsibility for the cared for. Sometimes respite allowed carers to escape the confines of their homes. For others, who normally had to take the cared for with them wherever they went, being able to leave them with a care worker allowed them to relax, not rush and even enjoy routine tasks such as visiting the supermarket. If their cared for benefitted from and enjoyed the company of the care worker, carers were particularly positive about respite. In fact, some carers focussed primarily on the necessity of the cared for to enjoy respite. In this way our research has highlighted that carers often do not see respite as a service for them alone and frequently see it as primarily for the cared for. This may be a reflection of the tendency for carers to prioritise the cared for’s needs over their own [33]. However, the fact that carers tend to use the time to do apparently routine tasks can be viewed in several ways. For example, for some carers respite gives them a sense of freedom allowing them to take pleasure in not always having the cared for with them. Others may have been unwilling to engage in activities designed specifically for their enjoyment perhaps because of a sense of guilt [21]. In a similar vein, it has been noted in research with stroke carers that some no longer feel able to take pleasure in leisure activities because they are not with the cared for [34]. Arguably, where possible, carers could be offered more time to allow them to use respite both for practical tasks and for recreational activities but the carers here varied in how they viewed respite and what they wanted from it. The perceived purpose and value of respite is therefore individual. However, it is essential that the carers trust the carer worker and believe that their cared for is at least happy and preferably benefitting from the situation.

Respite at home can have unforeseen benefits. Some care workers were valued for the time they spent with the carer, listening to them and bringing someone from outside their situations into their lives. This may well be a reflection of the social isolation experienced by so many carers [10] and may explain why some carers preferred to stay at home, rather than go out. Perhaps the care workers who were appreciated for their friendly lively characteristics when working with the cared for were also reducing the carers’ social isolation. Having regular care workers who can build up a relationship with the carer may also be particularly valuable.

For respite services to improve, both positive and negative feedback is essential but the relationships that develop in respite may result in carers offering little negative feedback. For example, a very friendly relationship with the care worker may mean that carers feel unable to criticise them. The perception here that care workers were usually kind people doing their best may also make voicing complaints difficult. In light of this, the boundaries and implications of the carer-care worker relationship should be considered and discussed in care worker training. The likelihood of complaining may be further reduced by the loss of autonomy associated with being a carer [34].

Understanding the impact of interventions, such as respite, to support carers is challenging as they take place in complex, often difficult, fluid social and health-related situations. Added to this, such interventions for carers are often part of a package of care making the impact of individual support services hard to isolate. Carers here were often caring for someone whose condition was deteriorating and in some cases the cared for were close to the end of their lives. For many carers such dynamic and often distressing situations might have a considerable negative impact on their well-being thus confounding positive effects of respite.

It is assumed that respite benefits carers by offering relief from caring, but the specific outcomes expected as a result of respite and the processes by which they are assumed to be achieved remain unclear. Our research suggests that benefits for carers may be individual. Therefore research using limited, specific outcome scales may not be meaningful for all carers, making it unlikely that evidence for the positive impact will be demonstrated. This may help explain why qualitative research that allows participants to describe outcomes relevant to them is more likely to suggest benefits for carers than quantitative research. We identified no clear differences in the benefits identified by the participants in our study in terms of their demographic characteristics or situations. However, most of our participants were caring for someone with dementia and perhaps with a larger sample with a greater diversity of carer characteristics and diagnoses, differences may have been identifiable.

Victor’s [22] review also drew attention to this contrast between the findings of qualitative and quantitative evaluations of interventions in general to carers. Overall, few quantitative studies report a significant positive impact on carers. In contrast, qualitative studies are more likely to report benefits. Victor [22] suggests that the measures used in quantitative research may lack validity and be insensitive to the complexity of outcomes such as carers’ emotional well-being. She also suggests that in the social situation of qualitative research interviews, carers may over-state the benefits of any intervention. However, the fact that most research investigating patient satisfaction with services reports the opposite, with lower satisfaction reported in qualitative research, for example [35], suggests that the social situation of the interview may not be the reason. Perhaps carers’ sense of powerlessness and the belief that they should be grateful for any support are also relevant here [34]. Whatever the reasons, the use of mixed methods in future research to evaluate such services may provide the most useful answers [36,37].

## Strengths and limitations of the study

The strength of this qualitative study lies in the depth of the carers’ descriptions of their experiences and perceptions of the impact of respite allowing us to suggest some relationships between, for example, carers’ perceptions of the care workers and the perceived impact of the respite on themselves and the cared for.

Concerns about the generalisability of qualitative research with small sample sizes are frequently raised. For this study, twelve carers were interviewed. The interviews focussed on perceptions of respite at home and were in considerable depth. So although relatively few carers took part in the study, arguably the strength of this study lies in the depth of the carers’ descriptions of their experiences. Conversely this therefore means we were unable to widen our discussion to include other forms of respite.

Perhaps because of the nature of the interview, carers may have over emphasised the benefits of any support but ambivalence about specifically criticising care workers was also clear. This may be because most care workers were thought to be very pleasant people doing their best, even if the carers were not totally happy with it. These carers were entrusting their cared for with people they knew little about and once they had agreed to accept respite, it may have been difficult to admit that the relationship was not working. Carers may have felt that complaining could lead to the end of the respite service for them.

A larger more diverse sample may have added to the findings. One specific limitation of this study is that there were few minority ethnic carers. The proportion of carers from minority ethnic groups is growing in the UK [38] but there is a dearth of research investigating how carers from minority ethnic groups perceive respite and would ideally like it provided. It has been reported [39] that some minority group carers respond positively to home based respite but that these carers also emphasised the importance of language and gender matching. Further work is needed here.

## Future research

Further research is needed that identifies more specifically what outcomes from respite might be expected for carers and their cared for. One way to do this may be to compare and contrast different types of respite in terms of the service providers’ aims and the carers’ and their cared for’s experiences and perceptions of the services. Using mixed methods may help understand why findings from qualitative and quantitative methods sometimes appear to conflict.

The current study highlighted the centrality of the cared for but there is little research investigating their experiences. Future research should pay greater attention to the diagnosis or condition of the cared for. This may not only have implications for the type of respite needed but also on the experience or qualifications of the care worker. It may also influence carers’ willingness to leave their cared for. Trust in the service provider is very important and more work is needed to understand the role of trust in different types of respite. Some research has suggested that carers and the cared for tend to prefer home based respite, for example [25], and the reasons for this need to be better understood especially given the sometimes ambiguous gain associated with care workers coming into the home.

Finally greater understanding of respite for carers of people with dementia is warranted. The complex and variable nature of the symptoms of dementia pose particular challenges for the provision of respite and requires highly trained and skilled care workers. Research that specifically investigates the experiences of the carers, the people with dementia and the care workers is called for.

## Conclusions

The value of respite lies partly in that it can allow carers a break from the often overwhelming, unrelenting sense of responsibility that many feel for their cared for, but our study has also demonstrated the complexity of providing respite services. Carers have to trust both the service provider overall and the individual care worker and the cared for must be comfortable and able to communicate well with the care worker. An additional important element is the care worker’s sensitivity to the carer’s and cared for’s needs and circumstances, but they must also be able to respond to any changes in the cared for’s condition or mood. Potential barriers to successful respite therefore include inadequate skills or training of care workers or poor matching of the cared for with the care worker. The challenges for the care worker must not be underestimated.

## Competing interests

The authors declare that they have no competing interests.

## Authors’ contributions

NG conceived of the study, led on its design, data analysis and drafted the manuscript. She also interviewed some participants. AM supported the design of the study and advised on the analysis. RH recruited and interviewed most of the participants and assisted in the analysis and drafting of the paper. All authors read and approved the final manuscript.

## Pre-publication history

The pre-publication history for this paper can be accessed here:

http://www.biomedcentral.com/1471-2318/12/42/prepub
